# The effect of counseling with a skills training approach on maternal functioning: a randomized controlled clinical trial

**DOI:** 10.1186/s12905-020-00914-w

**Published:** 2020-03-11

**Authors:** Fatemeh Karami Chamgurdani, Jennifer L. Barkin, Khalil Esmaeilpour, Jamileh Malakouti, Massimiliano Buoli, Mojgan Mirghafourvand

**Affiliations:** 1 Department of Midwifery, School of Nursing and Midwifery, Tabriz University of Medical Sciences, Tabriz, Iran; 2 Department of Community Medicine, Mercer University School of Medicine, Macon, GA USA; 3 Faculty of Education and Psychology, University of Tabriz, Tabriz, Iran; 4 Department of Midwifery, School of Nursing and Midwifery, Tabriz University of Medical Sciences, Tabriz, Iran; 5 Department of Psychiatry, 5University of Milan, Milan, Italy; 6 Social Determinants of Health Research Centre, Faculty of Nursing and Midwifery, Tabriz University of Medical Sciences, Tabriz, Iran

**Keywords:** Counseling, Skill training approach, Maternal functioning, Postpartum

## Abstract

**Background:**

The role of the mother can be deeply satisfying, but it is associated with many challenges including challenges during the postpartum period that may impede the optimal development of the infant. Therefore, the aim of the present study was to investigate the effects of counseling using the Skills Training Approach (STA) on postpartum maternal functioning.

**Methods:**

This randomized controlled trial was performed on 68 postpartum women who referred to health centers of Tabriz-Iran in 2019. Participants were assigned to one of two groups - either counseling or control through the block randomization method. The intervention group received four counseling sessions using the Skills Training Approach (STA). Before and two weeks after the completion of the intervention, the Barkin Index of Maternal Functionning (BIMF) was completed by the participants. The independent t-test and ANCOVA (Analysis of Covariance) was used to analyze the data.

**Results:**

There was no statistically significant difference between the two groups in terms of sociodemographic characteristics and the baseline scores of the BIMF and its domains (*p* > 0.05). Before the intervention, the mean (SD) total score of the BIMF in the intervention group was 73.1 (8.5) and in the control group, it was 71.6 (4.8). Post-intervention, the mean (SD) of the total score of the BIMF in the intervention group was 95.8 (11.8) and in the control group, it was 70.3 (4.5). Based on the ANCOVA test and after adjusting the baseline score, the mean total score of the BIMF was significantly higher in the intervention group than in the control group (Mean Difference (MD): 22.9; 95% CI: 18.2 to 27.6; *p* < 0.001). The post-intervention scores of all domains of the BIMF including self-care (MD: 3.8), infant care (MD: 2.0), mother-child interaction (MD: 4.8), psychological wellbeing (MD: 8.4), social support (MD: 4.0), management (MD: 6.8), and adjustment to new motherhood (MD: 3.2) were significantly higher in the intervention group compared to the control group (*P* < 0.001).

**Conclusion:**

In this study, counseling, using STA, was effective in improving maternal functioning in all of the domains. This intervention, aimed at skill-building, should be strongly considered where improved postpartum functioning is the goal.

**Trial registration:**

IRCT20120718010324N49. Registered 18 January 2019.

## Background

Being a mother is one of the most important roles in a woman’s life [1]. The process of pregnancy, delivery and early motherhood can lead to many serious psychological and social challenges for a woman and her family [2]. The birth of a child is a challenging life event that may affect the mother’s concept of self and her activities and opportunities [3]. The postpartum period or puerperium begins one hour after the birth of a child and lasts up to six weeks after delivery, during which the mother’s body returns to a non-pregnant state [4]. This is considered the transitional period for a new mother, newborn, and the family as a whole. Many complications can occur in this period, and if not recognized and quickly treated, may lead to physical discomfort, anxiety, low self-esteem, impaired quality of life [5], frustration, and postpartum depression (PPD) [6]. Asselmann et al. (2016) demonstrated that social support declined from prepartum to postpartum especially in women with comorbid anxiety and depressive disorders [7]. In recent years, the health of mothers and infants after delivery has been considered to be of special scientific interest. This is due to the fact that postpartum struggles can significantly impact the health of mothers and newborns, the development of the infant, (which is often due to the induction of childbirth or acute and chronic health problems), social roles and related coping, personal relationships, and mental disorders [8].

Maternal functioning in the postpartum period is considered optimal when the mother receives adequate social support, can care for herself [9] and her infant, is psychologically healthy (psychological well-being), participates in quality interaction with her infant, is able to manage her various responsibilities and adapts to those responsibilities over time [10]. Number of previous deliveries, social support, type of delivery, maternal and infant complications, and fatigue are among the many factors that can influence the postpartum experience [11]. The results of a literature review showed that the existing nursing literature on the concept of functional status after childbirth has focused on the physiological return to full function after delivery and asserts that mothers recover after six weeks of delivery. However, holistic recovery, which includes caring for the infant and oneself, engaging in social and occupational activities, and adjusting to role of motherhood may require several months to achieve [12].

Based on the Inventory of Functional Status After Childbirth (IFSAC), performance is dependent on a woman’s resumption of the roles she engaged in before giving birth [10]. The IFSAC is built on the premise that a full return to prenatal functional status is equivalent to healthy postnatal functioning [12]. By the developers’ calculations, the return to full functional status occurs three weeks to three months after delivery, and in a small number of mothers, between three to six months [13]. In one study, 57% of mothers were able to return to prenatal functional status six weeks after delivery. By 12 weeks, 76% had achieved this milestone [11]. Interestingly, many of these women mentioned that they had resumed their responsibilities-not because of their personal desires- but because of their commitment to the family and financial pressures [14].

Studies have shown that diminished maternal functioning has been associated with impaired infant growth [15] and decreased lactation [16]. Similarly, impaired function in the postpartum period may impede favorable cognitive development of the infant [15]. Therefore, evaluation of maternal functioning should be included in the postpartum work-up, as it provides valuable information about mother’s daily functioning and comfort level in juggling multiple role demands [17]. The studies conducted on Iranian mothers have shown that their functional status is at a moderate level [18, 19]. For a woman to fully achieve her postpartum functional status, she must resume most of the roles she had before she gave birth and birth can make it difficult for many women to make a full return to their functional status because of the changes often made in the lives of mothers [10]. Thus, there is a distinct need for interventions that target functional improvement via skill-building.

Counseling is a valuable and long-standing intervention and can be utilized to increase individual awareness and improve behavior [20]. Consultation is a process aimed at assisting a person in dealing with crisis and developing coping mechanisms. Active listening, mutual understanding, response, and targeted intervention is made possible through counseling and may be beneficial to the patient [21]. The Skills Training Approach (STA) is useful in promoting adaptability, which supports the client in coping with changing expectations. This approach is an educational, suitable, and feasible approach that can be conducted in all settings including public schools, family centers, outpatient clinics, general health centers, and family planning centers [22]. Skills training indirectly leads to profound ideological changes and prompts individuals to be aware of the lack of a skill in a particular area, It also leads to increased ability and skills via the use of audio and video tools, reading materials and other effective educational techniques [23]. The following learning activities comprise this approach (STA): (1) description of the purpose of the skill to be learned, (2) using the video-assisted modeling to demonstrate of the skill, (3) role-playing with the patients, (4) learning how to obtain the resources needed to do the skills, (5) performing in vivo exercises with the trainer’s assistance (outside of the training session itself), and (6) providing homework assignments to be conducted by the patients on their own in their natural environments with subsequent evidence that are completing their assignments [24, 25]. This method has been used to prevent smoking [26], manage autism [27] and schizophrenia [28], and improve the efficiency of psychiatric rehabilitation [29]. In all of the above studies, this approach was effective.

In studies in which the IFSAC was used, maternal functional status was found to be associated with maternal depression and low self-efficacy in the postpartum period [18], high levels of anxiety in women after childbirth [30], impaired infant development [15] and lactation difficulties [16]. However, only one research team in Iran has evaluated the impact of education on maternal functioning [24], and the IFSAC was used rather than the Barkin Index of Maternal Functioning (BIMF). The BIMF is a more recently developed and patient-centered measure of postpartum functioning [14]. In the present study, we aimed to examine the effect of counseling, using the Skills Training Approach, on postpartum maternal functioning as measured by the BIMF. This is a step toward improving maternal performance through skills-based interventions. The ultimate goal is to improve the health of mothers, their infants and the family unit as a whole by equipping mothers with the skills needed to navigate the complicated terrain of new motherhood.

## Methods

This research study is a randomized controlled clinical trial (RCT), using two parallel groups with 68 women who were referred to Tabriz-Iran health centers. Inclusion criteria included: A maternal functioning (as measured by the BIMF) score lower than 80, having 1 or 2 children, a competent mother (self-reported), maternal willingness to attend counseling sessions, lack of neonatal anomalies (including physical and mental disabilities), infant was full-term and healthy, the mother possesses at least secondary school or higher education, and natural postpartum period until admission (not having problems such as bleeding, lactation, infection and etc.). Exclusion criteria included: a post-partum depression score equal to or higher than 13 using the Edinburgh Postpartum Depression Scale (EPDS) [31], being unable or unsure of the ability to attend all counseling sessions, plans to move to relocate, presence of cardiovascular disease, high blood pressure, liver disease or other chronic diseases (as reported by the participant), neuropsychiatric diseases, or having recent calamities (as reported by the mother), and hospitalization of the infant.

### Sample size calculation

The sample size was determined according to the findings in Barkin et al.’s (2016) study [32], and by using the G-power software. Taking into account m_1_ = 80 (mean maternal functioning score), and assuming a 20% increase in the mean maternal functioning score resulting from the intervention (m_2_ = 96), sd_1_ = sd_2_ = 17, Two-sided α = 0.05, power = 95%), the desired sample size for each group was calculated to be 31. Factoring in potential 10% attrition, the final desired sample size was 34 for each group. In total, 68 women were selected for the counseling and control groups, satisfying the calculated sample size requirements.

### Recruitment

Subsequent to obtaining permission from the ethics committee of the Tabriz University of Medical Sciences (ethics code: IR.TBZMED.REC.1397.789) and registering the study at the Iranian Center for Clinical Trials (IRCT20120718010324N49), the research team began the recruitment process. Tabriz has 85 health centers and sampling was conducted at 13 health centers of varying levels of socioeconomic status. The researcher visited the centers and selected primiparous and second -birth mothers who gave birth in the three weeks prior, according to their health records. The selected women were invited to refer to an assigned health center during a telephone call. With in this intial conversation, they also received a brief explanation of the research and its importance. After mothers connected with their assigned health center, those who were interested in participating in the study were pre-registered. More detailed information was then provided on the goals, importance, and benefits of participation in the study as well as the implementation stages of the research. If interested, eligible, and prepared to participate in regular and continuous sessions, a written consent form was completed by the mothers. They also completed the BIMF [33] and EPDS [31] questionnaires. If they received a score of less than 13 on the EPDS, and a score below 80 on the BIMF, they were included in the study at which point other questionnaires such as the sociodemographic and obstetrics characteristics questionnaire were also completed. Two weeks after the last counseling session,a post-test questionnaire including the BIMF, was completed by participants in both the counseling and control groups.

### Randomization/group assignment

Participants were assigned to either the counseling or control groups using stratified block randomization (based on the number of deliveries and type of delivery) with the size of blocks being 4 and 6 and a 1:1 assignment ratio. To conceal the assignment, the type of intervention was written on a sheet of paper and placed inside matted, numbered envelopes. The envelopes were arranged according to the entry of the participants to the intervention, and the type of group was revealed.

### STA intervention

For the intervention group, counseling was provided using the STA. Accordingly, four counseling sessions were conducted at intervals of one week and for four consecutive weeks, from the fourth week after delivery with groups of 4 to 6 people, at the selected health centers. The duration of each session was on average 60 to 90 min. In all counseling sessions, we utilized the principles and techniques of counseling to achieve effective communication. When used appropriately, application of these core principles foster an atmosphere of respect and sincerity. They also promote patient self-confidence and provide a platform for open, honest discourse during group conversations. The specific skills were taught to the participants each week and practiced by them throughout the week; the skills were also practiced in the following session. In all sessions, video-assisted modeling and role-playing were employed. A general layout of the STA curriculum is as follows:

- **First session:** Initially, the group members were introduced to each other and the study objectives were explained. Self care and infant care were the two featured skills in this first session. At the end of this session, an educational booklet and the researcher’s phone number was distributed to the participants. The content of booklet was similar to topics discussed in the STA sessions.
- **Second session:** The previous session assignments were retrieved from the participants at the outset. The focus of the second session was mother-child interaction and maternal psychological wellbeing.
- **Third session:** The previous session assignments were retrieved from the participants and the skills of social support, physical, mental, and care management and adjustment were focused upon and practiced.
- **Forth session:** The previous session assignments were retrieved and the evaluation of participants knowledge was conducted.

The control group received only routine postpartum care including obtaining medical history (vaginal bleeding and discharge, urinary complaints, psychological status), clinical examinations (vital signs, breasts, abdomen, eyes, etc), assessing (nutrition, breastfeeding, family planning) and nutritional supplements (ferrous tables, multivitamins) [34].

### Data collection tools

#### Sociodemographic and clinical characteristics

The sociodemographic and clinical characteristics questionnaire includes questions about the age of the mother and her husband, the number of children, the gender of the infant, the level of education of the woman and her husband, the employment status of both spouses, income adequacy (adequate/inadequate), delivery type (vaginal/cesarean section), level of support of the husband and the family, and whether the pregancy was planned or not (Table 1). In order to determine the validity of the sociodemographic characteristics questionnaire, content and face validity were evaluated. In short, the questionnaire was distributed to ten experts and, after collecting their feedback, the required modifications were made..

**Table 1:** Sociodemographic characteristics of participants by study groups

Variable	Counselling group (*n* = 34) *n* (%)	Control group (*n* = 34) *n* (%)	*P*-value
**Age**^**#**^	29.8 (5.4)	29.1 (4.7)	0.601^*^
**Husband age**^**#**^	33.6 (5.3)	33.4 (3.5)	0.830^*^
**Child number**			0.808^†^
One	15 (44.1)	16 (47.1)	
Two	19 (55.9)	18 (52.9)	
**Child sex**			0.628^†^
Girl	16 (47.1)	18 (52.9)	
Boy	18 (52.9)	16 (47.1)	
**Job**			0.356^‡^
Housewife	33 (97.1)	30 (82.2)	
Employed	1 (2.9)	4 (11.8)	
**Husband’s job**			0.063^†^
Worker	9 (26.5)	4 (11.8)	
Employee	6 (17.6)	13 (38.2)	
Shopkeeper	5 (17.4)	9 (26.5)	
Others	14 (41.2)	8 (23.5)	
**Education**			0.171^**§**^
Secondary school	4 (11.8)	2 (5.9)	
High school	2 (5.9)	2 (5.9)	
Diploma	18 (52.9)	14 (41.2)	
Academic	10 (29.8)	16 (47.1)	
**Husband’s education**			0.259^**§**^
Elementary	2 (5.9)	0	
Secondary school	4 (11.8)	1 (2.9)	
High school	3 (8.8)	2 (5.9)	
Diploma	14 (41.2)	14 (41.2)	
Academic	11 (32.4)	17 (50.0)	
**Income**			0.149^**§**^
Completely enough	3 (8.8)	8 (23.5)	
Somewhat enough	30 (88.2)	25 (73.5)	
Not enough	1 (2.9)	1 (2.9)	
**Husband’s support**			0.190^†^
Too much	11 (32.4)	6 (17.6)	
Much	12 (35.3)	10 (29.4)	
Medium	7 (20.6)	8 (23.5)	
Low	4 (11.8)	6 (17.6)	
Very little	0	4 (11.8)	
**Family’s support**			0.064^‡^
Too much	12 (35.3)	9 (26.5)	
Much	15 (44.1)	8 (23.5)	
Medium	4 (11.8)	7 (20.6)	
Low	3 (8.8)	5 (14.7)	
Very little	0	5 (14.7)	
**Unwanted pregnancy**			0.604^†^
Yes	12 (35.3)	10 (29.4)	
No	22 (64.7)	24 (70.6)	

# Mean (Standard deviation)

§ Chi-square for trend test * Independent t-test ‡ Fisher’s exact test † Chi-square test

#### Maternal functioning

In this study, the BIMF was used to evaluate maternal postpartum functioning. The questionnaire was developed by Barkin et al. in 2010, includes 20 items, and incorporates seven domains of maternal functioning: self-care (items 2, 11, 13), infant care (items 12, 14), mother-child interaction (items 4,5,15), maternal psychological well-being (items 1, 2, 3, 5, 7, 10, 11, 16, 18, 20), social support (items 6, 8, 9), management (items 7, 11, 13, 14, 17, 18), and adjustment (items 17, 19); several items relate to more than one conceptual domain. The items are scored from 0 to 6, and the total score ranges from 0 to 120, with larger scores representing higher levels of functioning. The BIMF is used to measure maternal functioning during the 12–18 months after delivery and can potentially be used into the early toddler years [17]. The BIMF was chosen as the primary outcomes measure for the present study as it 1) was designed based on the experiences of women who had recently given birth, 2) is concise and validated, and 3) does not penalize women for reprioritizing after childbirth. Additionally, the mother’s psychological well-being is factored into the BIMF; this construct is not assessed within the IFSAC. Another attractive feature of the BIMF is that it was designed to be applicable in both research and clinical settings and takes only 5–10 min to complete [14]. In a separate language assessment, investigators found that the BIMF’s 20 items were comprehensible and clearly worded [33]. In this study, Cronbach’s alpha coefficient was 0.88 (indicating good internal consistency) and the Intra Correlation Coefficient (ICC) was equal to 0.85 [35].

#### Depressive symptoms

The EPDS is one of the most effective and established tools for measuring postpartum depression [33, 36]. The scale contains 10 multiple choice questions that address concepts such as feelings of overwhelm, anxiety, the inability to experience joy/pleasure, sadness, and thoughts of self-harm. Each item can take on values from 0 to 3 and the total score ranges from 0 to 30. A score of 13 or more is considered to be a significant case of postnatal depression, while scores between 10 and 12 represent borderline depression and 0 to 9 represents absence of depression. In Montazeri et al’s (2007) study [31], the Iranian version of this questionnaire was validated and is considered an acceptable instrument for measuring postpartum depression. The Cronbach’s alpha was reported as 0.77 and the test-retest reliability was 0.8, in the present study.

#### Analytic strategy

Upon the completion of data collection, analysis was conducted using SPSS version 24. The normality of quantitative variables was tested using the K-S test and all were normally distributed. In order to examine the consistency of the two groups in terms of sociodemographic characteristics, the chi-square, chi-square for trend, Fisher’s exact, and independent t-tests were conducted. In order to compare maternal functioning between the two groups pre-intervention, the independent t-test was used. At post-intervention, the ANCOVA test with baseline control was employed. A *p*-value < 0.05 was considered statistically significant.

## Results

**Fig. 1:**
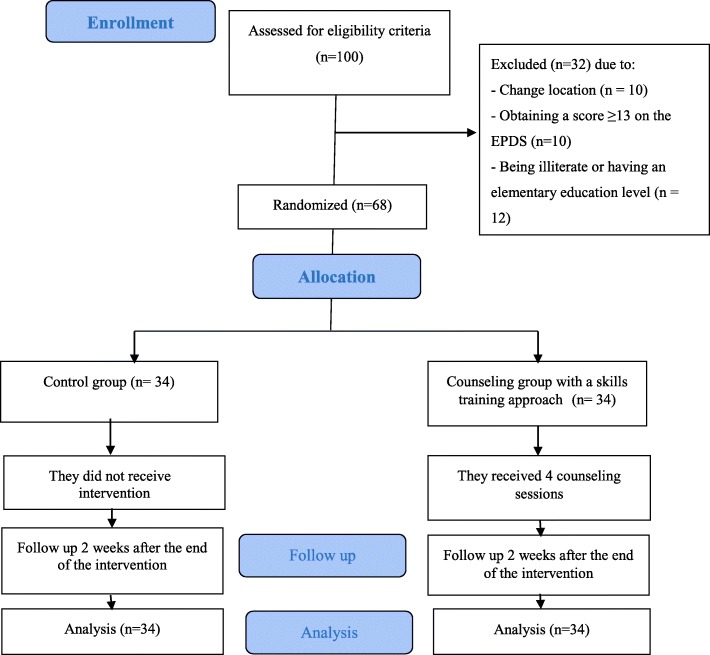
Flowchart of the study

This study began in February 2019 and ended in May 2019, covering a four month timespan. In the first month, the validity and reliability of data collection tools was assessed and confirmed. In the second month, recruitment of participants was conducted in health centers. In the third month, the counselling sessions were held for the intervention group. Two weeks after the completion of STA training, post-intervention questionnaires were completed and data was enterd into SPSS for the purposes of analysis. A total of 100 women were recuited from the health centers and evaluated based on eligibility criteria. Thirty two women were excluded from the study due to: changed location (*n* = 10), obtaining a score equal to or higher than 13 on the EPDS (*n* = 10) and/or being illiterate/having only completed elementary level education (*n* = 12). Sixty eight women were eligible for inclusion in the study, and were assigned to either counseling with STA or the control group. All women in the counseling and control groups were followed up until the end of the study and there was no drop out (Fig. 1).

The counseling and control groups were not significantly different in terms of baseline sociodemographic and clinical characteristics. The mean (SD) maternal age in the counseling group was 29.8 (5.4) and in the control group it was 29.1 (4.1). The mean (SD) age of the spouse was 33.6 (5.3) in the counseling group and 33.4 (3.5) in the control group. Most of the mothers in both groups were housewives (97.1% in the counseling group and 82.2% in the control group) and about one-third of their spouses were self-employed (41.2% in the counseling group and 23.5% in control group). More than half of the participants in both groups had two children and about half of them in both groups had daughters (47.1% in the counseling group and 52.9% in the control group). About one-third of the spouses in the counseling group (32.4%) and half in the control group (50%) had a higher education degree. The majority of women in both groups (88.2% in the counseling group and 73.5% in the control group) had a moderate economic status. About one third of women in both groups (35.3% in the counseling group and 29.4% in the control group) had an unwanted pregnancy. About a third of the participants in both groups (35.3% in the counseling group and 29.4% in the control group) enjoyed abundant husband’s support (Table 1).

The mean total BIMF score (SD) before intervention in the group who received counseling was 73.1 (8.5) and in the control group it was 71.6 (4.8); there was no statistically significant difference between the two groups (*P* = 0.383). Post-intervention, the mean total score (SD) of the BIMF in the counseling group was 95.8 (11.8) and in the control group it was 70.3 (4.5). Based on the ANCOVA test, and after adjusting for the baseline score, the mean score of the BIMF was significantly higher in the counseling group than in the control group (Mean Difference (MD): 22.9; 95% CI: 18.2 to 27.6; *p* < 0.001).

In summary, there was no significant difference between the two groups related to the seven BIMF domains before the intervention (*p* > 0.05). However, the post intervention scores of all BIMF domains including self-care (MD: 3.8; 95% CI: 2.8 to 4.8), infant care (MD: 2.0; 95% CI: 1.4 to 2.6), mother-child interaction (MD: 4.8; 95% CI: 3.9 to 5.7), psychological wellbeing (MD: 8.4; 95% CI: 6.8 to 9.9), social support (MD: 4.0; 95% CI: 3.1 to 4.8), management (MD: 6.8; 95% CI: 5.1 to 8.4), and adjustment (MD: 3.2; 95% CI: 2.4 to 3.9) were significantly higher in the intervention group compared to the control group (*P* < 0.001) (Table 2).

**Table 2:** Comparison of maternal functioning mean score between groups

Variable	Counselling (*n* = 34) Mean (SD^‡^)	Control (*n* = 34) Mean (SD^‡^)	Mean difference (95% Confidence Interval)	*P*-Value
**Total score of maternal functioning** (Score range: 0 to 120)				
Before intervention	73.1 (8.5)	71.6 (4.8)	1.5 (−1.9 to 4.8)	0.383^*^
Two weeks after the end of the intervention	95.8 (11.8)	70.3 (4.5)	22.9 (18.2 to 27.6)	< 0.001^**†**^
**Self-care** (Score range: 0 to 18)				
Before intervention	11.0 (2.8)	12.2 (1.7)	− 1.2 (−2.3 to −0.1)	0.041^*^
Two weeks after the end of the intervention	14.8 (2.2)	11.4 (2.1)	3.8 (2.8 to 4.8)	< 0.001^†^
**Infant care** (Score range: 0 to 12)				
Before intervention	9.5 (2.9)	8.6 (1.6)	0.9 (−0.2 to 2.1)	0.105^*^
Two weeks after the end of the intervention	10.8 (1.4)	8.5 (1.5)	2.0 (1.4 to 2.6)	< 0.001^†^
**Mother-child interaction** (Score range: 0 to 18)				
Before intervention	10.3 (3.1)	10.7 (1.6)	−0.4 (−1.6 to 0.8)	0.520^*^
Two weeks after the end of the intervention	15.1 (2.2)	10.5 (1.7)	4.8 (3.9 to 5.7)	< 0.001^†^
**Psychological well-being** (Score range: 0 to 60)				
Before intervention	35.6 (5.6)	34.3 (3.9)	1.3 (−1.0 to 3.7)	0.261^*^
Two weeks after the end of the intervention	43.3 (3.3)	34.7 (3.2)	8.4 (6.8 to 9.9)	< 0.001^†^
**Social support** (Score range: 0 to 18)				
Before intervention	10.5 (3.3)	9.8 (2.3)	0.7 (−0.7 to 2.1)	0.311^*^
Two weeks after the end of the intervention	15.1 (2)	10.9 (1.6)	4.0 (3.1 to 4.8)	< 0.001^†^
**Management** (Score range: 0 to 36)				
Before intervention	19.9 (3.7)	20.3 (2.95)	−0.5 (−2.1 to 1.2)	0.566^*^
Two weeks after the end of the intervention	26.6 (4.5)	19.97 (2.6)	6.8 (5.1 to 8.4)	< 0.001^†^
**Adjustment** (Score range: 0 to 12)				
Before intervention	7.3 (2.8)	7.79 (2.1)	−0.5 (−1.7 to 0.7)	0.440^*^
Two weeks after the end of the intervention	10.5 (1.4)	1.95 (7.5)	3.2 (2.4 to 3.9)	< 0.001^†^

^†^ ANCOVA test with adjusting the baseline score ^*^ Independent t-test ^‡^ Standard Deviation

## Discussion

The results of the present study show that counseling with STA led to improved overall maternal functioning, heightened performance in each of the BIMF domains. The results are impressive both in terms of the magnitude and statistical significance; the average BIMF score in the intervention group was over 25 points higher (post-intervention) than the mean of the control group and the result was highly statistically significant (*p* < 0.001).

The only other study conducted in Iran focused on maternal functioning was conducted by Bagherinia et al. in2017. The study was focused on determining the effect of an educational package on functional status and maternal self-confidence in primiparous women in the postpartum period. The study included 136 participants who were randomly assigned to one of two groups and the main outcome was the IFSAC. The intervention group consisted of 68 primiparous women referred to health centers in Tabriz-Iran for postpartum care. The intervention group received instruction in the form of face-to-face education, via telephone and a through an educational booklet. The control group included 68 primiparous women from the same health center. Data were collected through a sociodemographic questionnaire, the IFSAC, and the Lips Maternal Self-confidence Score (LMSCS), which was completed before the intervention and six weeks after the intervention. The results of the study showed that education had a positive effect on women’s self-esteem and, likewise, affected maternal functioning in a positive manner [19].

Kurdi et al. (2014) aimed to determine the effect of an educational program during and after pregnancy on primiparous mothers with unplanned pregnancies. Educational sessions included three group training sessions at 34, 35, and 36 weeks gestation and an individual training session on the 1st day after delivery. These training sessions included demonstrating behaviors of attachment to the fetus using dramatic techniques, with a doll and a CD. The training CD containing maternal role-play scenarios and a training manual focused on attachment to the fetus and newborn, general infant care, and breastfeeding was prepared by the researcher and given to the participants for home use. Results indicate that education led to increased maternal role attainment and maternal role satisfaction after childbirth [37]. The results of this study corroborate the findings of the aforementioned studies.

A study by Gurkan et al. (2017) was conducted with the aim of determining the effect of an antenatal training program on postpartum functional status and depressive symptoms in pregnant women referred to the prenatal care center at a hospital in Istanbul. The results showed that pre-pregnancy education did not reduce depressive symptoms and improve functional status after delivery. The results of this study are not consistent with the present study, perhaps because of the difference in the period of the education provision (pre-pregnancy period in the study of Gurkan et al. and postpartum period in this study), the use of different tool from the present study (IFSAC in the Gurgan study and the BIMF in this study), and/or a different study design (non-random in the Gurgan study and randomized study in this study) [38].

One of the domains of maternal functioning is (maternal) psychological well-being [10]. Given that drug interventions may come with unwanted side effects [39, 40], and potentially stigma, non-pharmacological psychological interventions aimed at skill-building may be particulary useful; the Skills Training Approach fits this description. Based on the obtained results, and the results of several other studies, it can be concluded that counseling and skills training interventions, have the potential to play an important role in maternal functional improvement after delivery. Therefore, we may have the ability to mitigate many mother and infant difficulties by enhancing maternal functioning through counseling with the skills training approach.

The strengths of the present study include the sampling scheme which included health centers with varied socioeconomic characteristics and no attrition. The strengths that characterize good clinical trial practice are also present, such as randomized assignment and concealment of the treatment assignment to avoid bias. Considering the type and number of childbirth cases in the present study, stratification was employed. Therefore, the study can be generalized for vaginal or cesarean. or to mothers experiencing their first or second childbirth. Another strong point of the present study was the choice of the BIMF as the primary outcomes measure. Among other desirable traits, such as brevity and concise item wording, the BIMF evaluates greater dimensions of compatibility and welfare with psychological well-being, management, social support, mother-child interaction and adjustment.

Considering the fact that this study was done on mothers without depression and with at least a secondary school education, this study cannot be generalized for women with depression, illiterate persons, or those with little education. Also, since this study was conducted in urban health centers, we have to be mindful to not assume that the study findings apply to rural residents. However, considering the fact that women in rural areas have greater needs for training programs and face additional challenges regarding access to care, it is recommended that a similar study be done in this region.

## Conclusions

Findings from the present study demonstrate that counseling with STA resulted in improved maternal functioning in all domains. Mothers have vital and often multi-dimensional roles including spouse, mother, daughter, worker, and citizen. As a result, they are faced with complex challenges, including infant feeding difficulties and sleep deprivation and require addditional support in order to function optimally (Barkin et al., 2014) [41]. Hence, we should pursue and invest in therapeutic approaches that positively impact maternal functioning through skills training, conducted by experts. Ware, Kosinski, and Keller (1996) [42] aptly state that, “*the goal of medical care for most patients today is to obtain a more “effective life” and to preserve functioning and well-being.” This statement is insightful and in sync with the skills training approach and the results observed within this study. An additional and important point is that in order to identify women who may benefit the most from therapies such as STA, healthcare providers must first screen new mothers for maternal functioning and perinatal mood and anxiety disorders* [43]*.*

## Data Availability

The datasets used and/or analyzed during the current study are available from the corresponding author on reasonable request.

## Abbreviations

95% CI: 95% Confidence Interval
ANCOVA: Analysis of Covariance;
BIMF: Barkin Index of Maternal Functionning
EPDS: Edinburgh Postpartum Depression Scale
ICC: Intra Correlation Coefficient
IFSAC: Inventory of Functional Status After Childbirth
LMSCS: Lips Maternal Self-confidence Score
MD: Mean Difference
RCT: Randomized Controlled Trial
SD: Standard Deviation
STA: Skills Training Approach
STA: Skills Training Approach
